# Effects of Ramadan fasting on glycemic control and lipid profile in type 2 diabetes mellitus: a systematic review and meta-analysis of clinical studies

**DOI:** 10.12701/jyms.2026.43.52

**Published:** 2026-08-05

**Authors:** Diana Mazaya Atsarina, Rachma Purnama Sari, Lisa Lestari, Ridwan Alfatah, R. Prindjati Prakasa

**Affiliations:** Faculty of Medicine, Universitas Islam Negeri Walisongo, Semarang, Indonesia

**Keywords:** Fasting, Glycemic control, Intermittent fasting, Lipids, Type 2 diabetes mellitus

## Abstract

**Background:**

Fasting is believed to confer metabolic health benefits; however, evidence regarding its effectiveness in patients with type 2 diabetes mellitus (T2DM) remains inconsistent. This study aimed to evaluate the effects of Ramadan fasting (RF) on the glycemic control, lipid profiles, and safety of patients with T2DM.

**Methods:**

PubMed/MEDLINE, Web of Science, Scopus, ScienceDirect, and the Cochrane Library were searched for clinical studies published until March 2026. Glycemic and lipid outcomes were pooled using a random-effects model and reported as mean differences (MDs) with 95% confidence intervals (CIs). The proportions of hypoglycemia and hyperglycemia were pooled using random-effects meta-analysis.

**Results:**

Thirty-four clinical studies involving 11,925 adults with T2DM were included. RF significantly improved fasting blood glucose (MD, –11.45; 95% CI, –17.48 to –5.42; *p*<0.01), glycated hemoglobin (MD, –0.46; 95% CI, –0.65 to –0.27; *p*<0.01), low-density lipoprotein (MD, –6.31; 95% CI, –11.81 to –0.81; *p*=0.02), and triglycerides (MD, –15.16; 95% CI, –25.97 to –4.34; *p*=0.01). No noteworthy improvements were observed in total cholesterol (MD, –9.38; 95% CI, –19.97 to 1.20; *p*=0.08) and high-density lipoprotein (MD, 0.81; 95% CI, –1.45 to 3.08; *p*=0.48). Substantial heterogeneity (*I*^2^=91%–97%) was observed across all outcomes. The pooled proportions of hypoglycemia and hyperglycemia were 6% (95% CI, 3%–10%) and 7% (95% CI, 0%–21%), respectively, with substantial heterogeneity (*I*^2^>96%).

**Conclusion:**

RF was associated with an average reduction in glycemic and certain lipid parameters, but hypoglycemia and hyperglycemia remained non-negligible risk factors. These findings should be interpreted as context-dependent associations across heterogeneous populations, rather than as a uniform effect of RF. Individualized monitoring and appropriate therapeutic adjustments are essential during RF.

## Introduction

Type 2 diabetes mellitus (T2DM) is a major global public health challenge. This is because the prevalence of this chronic and progressive metabolic disorder, characterized by hyperglycemia and insulin resistance, continues to increase rapidly, affecting 445 million people worldwide in 2020 [1], and contributing substantially to cardiovascular morbidity, microvascular complications, and mortality [2,3]. Although not universal, T2DM is commonly accompanied by abnormal lipid metabolism [4]. In patients with T2DM, suboptimal glycemic control and an unfavorable lipid profile play pivotal roles in accelerating vascular damage, increasing the risk of severe complications and worsening long-term prognosis [5-7]. Despite remarkable advances in pharmacological therapies for T2DM management, achieving and maintaining optimal glycemic and lipid control remains challenging for most patients, with the prevalence of poor glycemic control ranging from 45% to >90% [8]. Consequently, attention has increasingly shifted toward non-pharmacological interventions that may complement conventional therapies to achieve more comprehensive T2DM management.

One phenomenon that has attracted considerable attention and is highly relevant for investigation is fasting, particularly Ramadan fasting (RF). RF is a form of intermittent fasting observed annually by millions of Muslims worldwide for 1 month. During this period, individuals abstain from eating, drinking, intravenous fluid therapy, smoking, and oral medications from dawn until sunset [9]. Although some patients are medically exempt from fasting, the majority choose to observe it for spiritual and cultural reasons [10]. From a clinical perspective, RF is not merely a religious practice, but also a real-world model of intermittent fasting that may influence metabolic profiles associated with the risk of chronic diseases, including T2DM [11-13]. Changes in meal timing, sleep patterns, physical activity and exercise, caloric intake, and medication schedules during RF render its metabolic effects particularly complex and warrant further investigation.

RF may provide metabolic benefits for patients with T2DM by reducing energy intake, enhancing lipid mobilization, improving insulin sensitivity, and altering the body’s metabolic rhythm, all of which may contribute to improvements in blood glucose levels and lipid profiles [14-16]. However, these effects are not always consistent. In some patients, RF may increase the risk of dehydration, hypoglycemia, sleep disturbances, impaired cognitive performance, and mood changes [17,18]. Furthermore, these outcomes are likely influenced by multiple factors, including each patient’s baseline clinical status, type of antidiabetic medication used, dietary patterns during *suhoor* (the predawn meal) and *iftar* (the meal consumed at sunset to break the fast), physical activity levels, and daily fasting duration [19,20].

The clinical studies published to date have yielded inconsistent findings. Several studies have indicated improvements in glycemic control and lipid profiles after RF, whereas other studies have found no significant changes. Although several meta-analyses published over the past 5 years have attempted to statistically synthesize the effects of RF in patients with diabetes mellitus, the most recent meta-analyses focused exclusively on glycemic control [21], while another evaluated its effects in adolescents with type 1 diabetes mellitus (T1DM) [22]. Therefore, an updated systematic review and meta-analysis focusing not only on glycemic control, but also on lipid profiles and the incidence of glycemic safety events is warranted to address this research gap and provide more robust and quantitative evidence regarding the effects of RF in patients with T2DM. This study aimed to synthesize the pooled associations between RF and glycemic control, lipid profiles, and glycemic safety outcomes in patients with T2DM and to characterize the extent and potential sources of variability in these associations across studies, thereby providing a more nuanced, context-aware scientific basis for clinicians to guide patient education and offer individualized recommendations for those who wish to observe RF.

## Methods

### 1. Protocol and registration

The systematic review and meta-analysis protocol was registered in PROSPERO under the registration number CRD420261415862. To ensure comprehensive reporting, this study relied on a multistage selection process governed by the Preferred Reporting Items for Systematic Reviews and Meta-Analyses (PRISMA) 2020 recommendations [23].

### 2. Eligibility criteria

The inclusion criteria were: (1) clinical studies, including observational, cohort, and randomized or non-randomized controlled trials, involving patients with T2DM as the primary study population who observed RF; (2) studies including adult patients with T2DM, with or without comorbidities; (3) studies with either a control group comprising individuals without T2DM or no control group; (4) clinical studies reporting fasting blood glucose (FBG), glycated hemoglobin (HbA1c), total cholesterol (TC), low-density lipoprotein (LDL), high-density lipoprotein (HDL), and/or triglyceride (TG) levels; (5) studies reporting outcome data at a minimum of two time points, namely before RF and during the last week of or after RF; and (6) original articles published in peer-reviewed journals in English.

Studies were excluded if they: (1) included patients with T1DM, other types of diabetes mellitus, healthy individuals, or women who are pregnant; (2) did not report outcome data in a numerical format (e.g., data presented only as figures, diagrams, or graphs), thereby precluding data extraction; (3) evaluated RF in combination with interventions or medications other than antidiabetic agents that could potentially modify the effects of RF; or (4) were review articles, preclinical animal studies, unpublished manuscripts, preprints, conference abstracts, or editorials.

### 3. Information sources

The following electronic databases were systematically searched: PubMed/MEDLINE, Web of Science, Scopus, ScienceDirect, and the Cochrane Library. A manual search was also conducted to identify potentially relevant studies that were not retrieved through the electronic database search by performing citation tracking of the included studies using Google Scholar. Gray literature, including theses, dissertations, conference proceedings, preprints, and unpublished manuscripts, was not searched because a reproducible search strategy for these sources could not be standardized. Moreover, the literature from these sources does not undergo peer review and is more likely to have lower methodological quality [24].

### 4. Search strategy

The core search terms were developed by combining Medical Subject Headings (MeSH) terms and free-text keywords using Boolean operators: (“Ramadan fasting” OR “Ramadan intermittent fasting”) AND (“diabetes” OR “diabetes mellitus” OR “type 2 diabetes mellitus” OR “glycemic control” OR “blood glucose” OR “glycated hemoglobin” OR “lipid profile” OR “cholesterol” OR “LDL” OR “HDL” OR “triglycerides”). The search strategy was adapted to the syntax and indexing system of each database and is presented in Supplementary Table 1. The literature search and development of the search strategy were performed independently by two investigators (DMA and RPS). The following filters were used in the search: document type (article), language (English), and source type (journal). No restrictions on publication year were imposed; therefore, all eligible studies published from database inception to March 2026 were considered for inclusion.

### 5. Selection process

The study selection process comprised two main stages. Before screening, all records exported from the electronic databases in RIS and/or BibTeX format were imported into the Mendeley reference manager for automatic duplicate removal. In the first stage, the deduplicated records were screened based on their titles and abstracts, and studies deemed irrelevant to this meta-analysis were excluded. In the second stage, the full texts of potentially eligible studies were assessed against the predefined eligibility criteria. The entire selection process was documented using a PRISMA flowchart. Two authors (DMA and LL) independently selected the studies, and disagreements were resolved through consultation with a third author (RA) when necessary.

### 6. Data extraction

Data extraction was independently performed by two authors (RPS and RA) and validated by a third author (DMA). Any discrepancies identified during the process were resolved through in-depth discussions among the authors. Microsoft Excel 2016 for Windows (Microsoft Corp., Redmond, WA, USA) was used to extract the following data: study, country, participants, age, baseline T2DM status, and RF duration. In addition, data on hypoglycemia and hyperglycemia occurring during RF were extracted when reported; the extraction criteria and analytical approach for these outcomes are described in the adverse events subsection 10 below.

Baseline T2DM status was defined as the participants’ clinical status before the intervention. Eligible studies were required to clearly report whether the participants had T2DM and/or were receiving any type of antidiabetic medication. Two primary time points were extracted: (1) pre-RF data, defined as data collected before or immediately before the start of RF, and (2) post-RF data, defined as data collected during the last week of RF or after RF completion. The clinical outcomes analyzed included FBG (mg/dL), HbA1c (%), TC (mg/dL), LDL (mg/dL), HDL (mg/dL), and TG (mg/dL) levels.

For studies with multiple study arms or those involving patients with T1DM or control groups comprising individuals without T2DM or non-fasting populations, only data from the study arm(s) involving patients with T2DM who participated in RF were extracted. Similarly, for parallel-arm studies in which patients with T2DM who participated in RF received an additional intervention beyond RF and the control group consisted of patients with T2DM who only participated in RF, only the data from the control group were extracted.

### 7. Quality assessment

Two reviewers (RA and RPP) independently assessed study quality using the Quality Assessment Tool for Before-After (Pre-Post) Studies With No Control Group developed by the National Heart, Lung, and Blood Institute (NHLBI; https://www.nhlbi.nih.gov/health-topics/study-quality-assessment-tools). Although the original publications included observational studies, cohort studies, and randomized or nonrandomized controlled trials, only within-participant pre- and post-RF data from the RF arm(s) were extracted for quantitative synthesis. Consequently, all datasets included in the meta-analysis were analyzed as single-arm before-after comparisons. Therefore, the NHLBI before-after assessment tool was selected to evaluate the methodological quality of the synthesized evidence because all synthesized datasets represented within-participant pre-post comparisons [21]. This tool comprises 12 assessment criteria with each item rated as Yes, No, or Other (not available/not reported). Based on the ratings across all criteria, the overall study quality was classified as Good, Fair, or Poor. The two reviewers critically discussed each assessment item and overall quality rating to ensure consistency in their evaluations. Any disagreements arising during the assessment process were resolved through in-depth discussions and, when necessary, consultation with a third author (DMA) to reach a consensus.

### 8. Statistical analysis

Statistical analyses were performed to compare the clinical outcomes before and after RF. All clinical outcomes are expressed as means and standard deviations (SDs). Data reported as medians have been converted using the method proposed by Wan et al. [25]. Additional conversions were performed to derive SDs from standard errors (SEs) using the following formula: SD=√N×SE, where N is the sample size. Similarly, SDs were calculated from 95% confidence intervals (CIs) using the following formula: SD=√N×([CI_upper_-CI_lower_]/3.92) [26].

Studies reporting FBG levels in mmol/L were converted to mg/dL by multiplying the mean and SD by 18.018. For HbA1c levels reported in mmol/mol, the values were converted to % by multiplying both the mean and SD by 0.09148 and adding a constant of 2.152 to the converted mean. For the lipid profile outcomes, TC, HDL, and LDL values reported in mmol/L were converted to mg/dL by multiplying the mean and SD by 38.67, whereas TG values were converted using a factor of 88.57.

Given the expected variability in participants’ baseline characteristics, antidiabetic treatments, total RF duration, daily fasting duration, and geographic settings, pooled analyses were performed using a random-effects meta-analysis with the restricted maximum likelihood estimator and reported as mean differences (MDs) with 95% CIs. Statistical heterogeneity was assessed using the chi-square test, with *p*<0.10 indicating significant heterogeneity, and the *I*^2^ statistic, which was interpreted as low (<25%), moderate (25%–75%), or substantial (>75%) [27].

In the statistical software, post- and pre-RF data were entered as the treatment and control groups, respectively. A negative pooled effect estimate indicated a reduction in the outcome after RF, whereas a positive estimate indicated an increase. As only data from the T2DM arm(s) observing RF were extracted for pooling (see Subsection 6, Data extraction), the pre- and post-RF measurements were obtained from the same participants within each study and were therefore inherently paired. Additionally, because most of the included studies reported only summary statistics at pre- and post-RF time points and the SDs of within-participant changes and pre-post correlation coefficients were generally unavailable, pooled analyses were performed using the reported pre- and post-Ramadan summary data. Accordingly, MDs represent the difference between the post- and pre-RF group means. No imputation of within-participant correlation coefficients was performed because the required information was not consistently reported across the primary studies.

Leave-one-out sensitivity analysis was performed to assess the robustness of the findings by sequentially omitting each study and recalculating the remaining studies. Given the substantial variability among studies and wide variation in RF characteristics across different regions, exploratory subgroup analyses were also conducted to investigate the potential sources of heterogeneity. The subgroups were stratified according to the United Nations M49 geographic subregions (https://unstats.un.org/unsd/methodology/m49/), study quality, and fasting duration. The cutoff for fasting duration (<15 hours vs. ≥15 hours) was determined based on the distribution of fasting durations reported across the included studies. All statistical analyses were performed using Stata MP ver. 17.0 for Windows (StataCorp LLC, College Station, TX, USA).

### 9. Reporting bias

For meta-analyses that included at least 10 studies, publication bias was assessed using funnel plots by plotting the MD against its SE [28]. The funnel plots were visually inspected independently by two authors (RPS and RA). Any disagreements in interpretation were resolved through discussion, with consultation from a third author (DMA) when necessary to reach a consensus. In addition to visual inspection, formal statistical tests were performed to validate the funnel plot findings using the Begg test [29] and Egger test [30], with *p*<0.10 considered indicative of publication bias.

### 10. Adverse events

Data on hypoglycemia and hyperglycemia occurring during RF were extracted from all included studies that reported such data at the patient level, defined as the number of patients who experienced at least one episode of mild-to-severe hypoglycemia or hyperglycemia relative to the total number of patients. As the definitions of hypoglycemia and hyperglycemia varied across studies (e.g., differing blood glucose thresholds and monitoring methods), no attempt was made to harmonize these definitions, and this variability is acknowledged as a limitation.

Pooled proportions of hypoglycemia and hyperglycemia, with 95% CIs, were estimated separately using a random-effects meta-analysis of proportions with the DerSimonian-Laird estimator and the Freeman-Tukey double arcsine transformation. The pooled estimates were back-transformed to the original proportion scale for reporting. Heterogeneity was assessed using the chi-square test and the *I*^2^ statistic, applying the same thresholds as for the primary outcomes. All analyses were performed using the *metaprop* command [31] in Stata MP ver. 17.0 for Windows.

## Results

### 1. Study selection

After removing duplicate records, 1,166 articles were identified through database searching. During the first stage of screening, 1,037 articles were excluded because they were irrelevant to this systematic review, leaving 129 articles for full-text assessment. In the second stage, full-text evaluation resulted in the exclusion of 99 reports for the following reasons: unavailable full text, irrelevant objective, T1DM population, mixed diabetes population, gestational diabetes population, no pre-fasting baseline data, ineligible outcomes, noncontinuous outcome data, insufficient data for extraction, qualitative study, review article, report, preprint, or editorial. Thus, 30 studies were included from the database search.

Citation tracking identified an additional 12 records. Following full-text assessment, eight articles were excluded because of unavailable full text, insufficient data for extraction, ineligible outcomes, or language, resulting in the inclusion of four additional studies. Overall, 34 clinical studies [32-65] were included. The complete study selection process is illustrated in Fig. 1.

### 2. Study characteristics

A total of 34 clinical studies involving 11,925 adults with T2DM were included in this meta-analysis. These studies were conducted across several geographic subregions, including Northern Africa [32,33,44,48,51], Western Asia [35-39,41,43,46,47,49,50,57-60,63], Southern Asia [34,52-56,61], Southeastern Asia [62,65], Sub-Saharan Africa [40], and Western Europe [64]. In addition, two multicenter studies were conducted across multiple subregions, including (1) Western Asia and Northern Africa [42], and (2) Asia, Africa, and Europe [45]. Based on methodological quality assessment, 26 studies [32,33,36,37,40-57,59,61-63] were rated as Good, whereas the remaining eight studies [34,35,38,39,58,60,64,65] were rated as Fair (Table 1).

### 3. Ramadan fasting and glycemic control

The pooled random-effects analysis showed that RF significantly reduced FBG levels by 11.45 mg/dL (number of studies [*n*_studies_]=21; MD, –11.45; 95% CI, –17.48 to –5.42; *p*<0.01), although substantial between-study heterogeneity was observed (*I*^2^=94%, *p*<0.01) (Fig. 2A). A significant reduction in HbA1c levels of 0.46% was also observed after RF (*n*_studies_=33; MD, –0.46; 95% CI, –0.65 to –0.27; *p*<0.01); however, substantial heterogeneity was observed (*I*^2^=96%, *p*<0.01) (Fig. 2B).

### 4. Ramadan fasting and lipid profiles

The pooled analysis showed that RF was associated with a nonsignificant reduction in TC levels (*n*_studies_=17; MD, –9.38; 95% CI, –19.97 to 1.20; *p*=0.08) (Fig. 3A) and a nonsignificant increase in HDL levels (*n*_studies_=18; MD, 0.81; 95% CI, –1.45 to 3.08; *p*=0.48) (Fig. 3C), with substantial heterogeneity (*I*^2^=97%, *p*<0.01 and *I*^2^=96%, *p*<0.01, respectively). In contrast, RF was significantly associated with a reduction in LDL levels of 6.31 mg/dL (*n*_studies_=18; MD, –6.31; 95% CI, –11.81 to –0.81; *p*=0.02) (Fig. 3B) and a reduction in TG levels of 15.16 mg/dL (*n*_studies_=19; MD, –15.16; 95% CI, –25.97 to –4.34; *p*=0.01) (Fig. 3D), although substantial heterogeneity was observed for both outcomes (*I*^2^=91%, *p*<0.01 and *I*^2^=96%, *p*<0.01, respectively).

### 5. Subgroup analysis

Substantial heterogeneity was observed across all outcomes (*I*²>90%). Therefore, exploratory subgroup analyses were conducted based on geographic subregion, fasting duration, and study quality to investigate the potential sources of heterogeneity (Table 2). Because these analyses were exploratory, the findings should be interpreted with caution.

For FBG outcomes, heterogeneity decreased markedly among studies conducted in Western Asia (*I*^2^=46%, *p*=0.16) and those rated as Fair (*I*^2^=57%, *p*=0.13). The subgroup analysis also showed that the pooled effect estimates became nonsignificant for studies conducted in Northern Africa, studies with fasting durations of <15 hours and ≥15 hours, and studies of Fair quality. However, the *p*-value for the interaction indicated no significant differences in effect estimates across subgroups stratified by geographic subregion (*p*=0.57) or fasting duration (*p*=0.63), whereas a significant interaction was observed for study quality (*p*<0.01). These findings indicate that study quality may have contributed to the observed between-study heterogeneity.

For HbA1c levels, reduced heterogeneity was observed only among the studies conducted in Southeastern Asia (*I*^2^=0%, *p*=0.83). Although the overall effect estimates remained generally consistent across most subgroups, they became nonsignificant in studies from Southeastern and Southern Asia as well as in studies with fasting durations of <15 hours. Nevertheless, no significant subgroup differences were detected based on the geographic subregion (*p*=0.42), fasting duration (*p*=0.72), or study quality (*p*=0.81). These findings indicate that the substantial heterogeneity observed in HbA1c levels is unlikely to be explained by differences in geographic subregions, fasting duration, or study quality.

For TC levels, stratification by fasting duration substantially reduced heterogeneity among studies with fasting durations of ≥15 hours (*I*^2^=0%, *p*=0.70). Furthermore, the pooled effect estimates became statistically significant among studies conducted in Southern Asia and among those with fasting durations of ≥15 hours, although the number of studies in these subgroups was limited. Interestingly, studies from Northern Africa showed a nonsignificant increase in TC levels following RF. In addition, a significant interaction was observed across geographic subregions (*p*<0.01), suggesting that geographic subregions contributed to the substantial heterogeneity in this outcome.

For LDL levels, the pooled effect estimates became nonsignificant among studies conducted in Northern Africa, Southeastern Asia, and Western Africa, as well as among studies with fasting durations of <15 hours and those rated as Fair. Reduced heterogeneity was observed among studies conducted in Southeastern Asia (*I*^2^=0%, *p*=0.83) and Southern Asia (*I*^2^=0%, *p*=0.31), studies with fasting durations of ≥15 hours (*I*^2^=0%, *p*=0.81), and studies of Fair quality (*I*^2^=27%, *p*=0.43). However, a significant interaction was detected across geographic subregions (*p*=0.02), indicating that geographic subregions may be important contributors to the substantial heterogeneity observed in LDL levels.

For HDL levels, significant pooled effects were observed among studies conducted in Southern Asia and among those with fasting durations of ≥15 hours. Heterogeneity was reduced among studies conducted in Southeastern Asia (*I*^2^=0%, *p*=0.99) and Southern Asia (*I*^2^=0%, *p*=0.61), studies with fasting durations of ≥15 hours (*I*^2^=0%, *p*=0.87), and studies rated as Fair (*I*^2^=0%, *p*=0.99). However, the *p*-values for the interactions showed no significant subgroup differences based on geographic subregion (*p*=0.11), fasting duration (*p*=0.32), or study quality (*p*=0.70), suggesting that none of these factors likely accounted for the substantial heterogeneity.

Finally, for TG levels, reduced heterogeneity accompanied by a nonsignificant pooled effect estimate was observed among studies conducted in Southeastern Asia (*I*^2^=0%, *p*=0.55) and among studies with fasting durations of ≥15 hours (*I*^2^=0%, *p*=0.74). Heterogeneity was also reduced among studies from Northern Africa (*I*^2^=0%, *p*=0.84), and the pooled effect estimate became nonsignificant among studies of Fair quality. Furthermore, a significant interaction was observed only for fasting duration (*p*=0.01), whereas no significant interactions were found for geographic subregion (*p*=0.15) or study quality (*p*=0.83). These findings indicate that fasting duration may be an important contributor to the substantial heterogeneity observed in TG levels.

### 6. Sensitivity analysis

The analysis demonstrated that the pooled estimates for FBG, HbA1c, HDL, and TG levels were robust, as evidenced by the stability of both the effect estimates and *p*-values after the sequential exclusion of individual studies. By contrast, the pooled estimates for TC and LDL levels were less stable, with changes observed in both the effect estimates and *p*-values, suggesting that certain studies contributed to the instability of these findings. The results of these analyses are presented in Supplementary Fig. 1.

### 7. Publication bias

Visual inspection of the funnel plots suggested asymmetry for FBG (Fig. 4A), HbA1c (Fig. 4B), TC (Fig. 4C), and TG (Fig. 4F) outcomes, whereas the funnel plots for LDL (Fig. 4D) and HDL (Fig. 4E) outcomes appeared to be relatively symmetrical. Formal statistical tests were performed to validate the visual findings. No evidence of publication bias was detected for TC, LDL, HDL, or TG levels according to the Egger test (*p*=0.70, *p*=0.44, *p*=0.80, and *p*=0.84, respectively) or Begg’s test (*p*=0.36, *p*=0.83, *p*=0.61, and *p*=0.19, respectively). For FBG and HbA1c levels, evidence of publication bias was detected by the Egger test (*p*=0.02 and *p*=0.08, respectively) but not by Begg’s test (*p*=0.40 and *p*=0.19, respectively).

### 8. Adverse events

Data on hypoglycemia during RF were available from 18 studies [35,38,40-42,45,47,53-57,59-62,64,65]. The pooled proportion of patients experiencing at least one hypoglycemic episode was 6% (95% CI, 3%–10%) with substantial heterogeneity (*I*^2^=97%, *p*<0.01) (Fig. 5A). Data on hyperglycemia were available from six studies [42,47,54,57,64,65]. The pooled proportion was 7% (95% CI, 0%–21%), also with substantial heterogeneity (*I*^2^=97%, *p*<0.01) (Fig. 5B). Given the substantial heterogeneity and, for hyperglycemia, the wide 95% CI around the pooled estimate, these proportions should be regarded as an approximate summary of the reported safety profile of RF, rather than a precise estimate applicable to all patients with T2DM.

## Discussion

This meta-analysis, which included 34 clinical studies, evaluated the association between RF and glycemic control, lipid profiles, and glycemic safety outcomes in patients with T2DM. Because RF is a complex behavioral intervention rather than a standardized therapeutic exposure, its metabolic effects are expected to vary considerably across clinical settings. Therefore, the pooled estimates reported here should be understood as average associations across highly heterogeneous populations rather than a uniform or universal effect of RF.

This study found that RF was associated with significant improvements in FBG, HbA1c, LDL, and TG levels, whereas no significant effects were observed for TC or HDL levels. However, these primary findings should be interpreted considering several important methodological considerations. Although the pooled results were generally robust according to sensitivity analyses, the findings for TC and LDL were less stable, suggesting that a small number of studies exerted a disproportionate influence on the pooled effect estimates. Furthermore, publication bias was detected for FBG and HbA1c levels based on the Egger test, indicating that the possibility of small-study effects or preferential publication of favorable results cannot be excluded. It should be noted, however, that funnel plot asymmetry can also arise from substantial between-study heterogeneity rather than publication bias alone. Given the considerable heterogeneity observed across all outcomes in this meta-analysis, the observed asymmetry for FBG and HbA1c may partly reflect true clinical and methodological diversity among the included studies rather than selective publication bias per se. Most importantly, substantial heterogeneity (*I*²>90%) was observed across all outcomes, suggesting that the estimated effects were unlikely to be consistent across all study populations. Therefore, the pooled estimates should be interpreted as the average effect across highly heterogeneous clinical settings, rather than as a universal effect of RF in all patients with T2DM.

Although this meta-analysis demonstrated significant reductions in FBG and HbA1c levels, these findings should be interpreted with caution. A reduction in mean glycemic parameters at the population level does not necessarily reflect an optimal metabolic safety profile at the individual level. A pooled analysis of the available safety data indicated that 6% of patients experienced at least one hypoglycemic episode during RF, whereas 7% experienced at least one hyperglycemic episode. However, both estimates showed substantial heterogeneity (*I*^2^>90%). In addition, the definitions and monitoring methods varied substantially across studies, and safety profile outcomes were reported in only a subset of the included studies; therefore, the results should be interpreted cautiously. In contrast, several studies reported no adverse events, including hypoglycemia or hyperglycemia, whereas other studies did not provide data on these safety outcomes. This substantial variability suggests that glycemic responses during RF are likely influenced not only by the physiological effects of fasting itself, but also by patient characteristics, pre-Ramadan glycemic control, and the type of antidiabetic therapy used. Several studies indicated that hypoglycemic events occurred more frequently among patients receiving insulin, either as monotherapy or in combination with oral antidiabetic agents, and among those treated with sulfonylureas [35,44]. Therefore, the reductions in FBG and HbA1c levels observed in this meta-analysis should not be interpreted as evidence that RF is universally safe for all patients with T2DM. These findings suggest that RF is associated with improved glycemic control in appropriately selected patients with well-controlled T2DM. However, these potential benefits should be carefully weighed against the persistent and variable risk of acute glycemic disturbances in certain patient subgroups.

This study explored the sources of the substantial heterogeneity through subgroup analyses. The findings indicate that the geographic subregion, fasting duration, and study quality may have contributed to the observed heterogeneity, particularly for FBG, TC, LDL, and TG outcomes. However, these findings should be interpreted with caution, because the number of studies within several subgroups was limited, rendering the pooled estimates susceptible to instability and reducing the statistical power to draw reliable conclusions. Moreover, the persistence of substantial residual heterogeneity indicates that other unexamined factors are likely to have contributed, including baseline glycemic control and lipid profiles, antidiabetic treatment regimens, medication dose adjustments during RF, caloric intake and meal composition during *suhoor* and *iftar*, physical activity, daily fasting duration, and variations in outcome definitions and measurement time points across the studies. Therefore, the subgroup analyses presented in this study should be regarded as exploratory and hypothesis-generating rather than as definitive evidence identifying the sources of heterogeneity.

A meta-analysis conducted by Elmajnoun et al. [21] that included only young adults with T2DM showed that RF significantly improved HbA1c and FBG levels. These findings remain consistent with those of the present meta-analysis, despite the broader sample that included adults with no upper age limit. However, our findings differ from those of an earlier meta-analysis by Tahapary et al. [66] that reported that RF improved FBG and HbA1c levels as well as TC, LDL, HDL, and TG levels. In contrast, the present meta-analysis did not find significant improvements in TC and HDL levels. This inconsistency may reflect shifts in study composition and the inclusion of more recent studies that may have had more heterogeneous baseline characteristics or smaller effect sizes, thereby weakening the previously reported beneficial signal on lipid profiles.

Fasting, including RF as a form of intermittent fasting, has been associated with significant clinical benefits for glucose and lipid metabolism in humans. However, the underlying biological mechanisms have not yet been fully elucidated [16,67,68]. Physiologically, the restriction of meal timing and energy intake during fasting may enhance tissue glucose utilization, promote glucose storage as glycogen, and maintain glucose homeostasis through the activation of gluconeogenesis and increased activity of key enzymes, such as phosphoenolpyruvate carboxykinase and glucose-6-phosphatase, to regenerate glucose when exogenous nutrient intake is reduced [68-70]. Furthermore, reduced energy intake during fasting may attenuate adiposity and insulin resistance through metabolic reprogramming, sustained reductions in insulin secretion, and increased activation of adenosine monophosphate-activated protein kinase, which plays a central role in improving insulin sensitivity, maintaining glucose homeostasis, and regulating hepatic lipid metabolism [71-73]. At a broader molecular level, fasting has also been associated with alterations in signaling pathways and regulatory proteins involved in glucose and lipid metabolism, DNA repair, circadian rhythm regulation, immune modulation, cytoskeletal remodeling, cognitive function, and enhanced autophagy [12].

Regarding lipid metabolism, fasting induces a metabolic switch that shifts energy utilization toward TG lipolysis, fatty acid oxidation, and ketone body production. Concurrently, increased expression of peroxisome proliferator-activated receptor (PPAR) alpha and PPAR gamma coactivator-1 alpha may promote fatty acid oxidation and apolipoprotein-A synthesis, which is associated with increased HDL levels, whereas reduced apolipoprotein-B expression and regulation of sterol regulatory element-binding protein-2 may decrease hepatic TGs, LDL, and TC [74,75]. Collectively, these mechanisms provide a plausible biological basis for the improvements in glycemic and lipid parameters observed during RF. Nevertheless, the magnitude of these effects is likely to depend on the clinical context, patient characteristics, and appropriate adjustment of antidiabetic therapy.

From a clinical perspective, these findings underscore that RF in patients with T2DM should not be regarded as a universally safe or inherently harmful practice. Rather, its suitability should be evaluated on an individual basis, according to each patient’s metabolic status and overall risk profile. The significant improvements observed in glycemic control suggest that some patients derive metabolic benefits from RF. However, the substantial between-study heterogeneity, reported occurrence of both hypoglycemia and hyperglycemia, and presence of publication bias warrant cautious interpretation of these findings. Consequently, decisions regarding RF should be guided by pre-Ramadan risk stratification, the assessment of glycemic control and comorbidities, type of antidiabetic therapy prescribed, and the patient’s ability to perform glucose monitoring and adhere to individualized dietary and medication adjustments. From a practical standpoint, these findings support the importance of structured pre-Ramadan education, individualized adjustment of antidiabetic regimens, and close glucose monitoring throughout the fasting period. Overall, the potential benefits of RF for glycemic control and lipid profiles can only be optimized when accompanied by a personalized, safety-oriented clinical approach.

This study had several limitations. First, all outcomes exhibited substantial heterogeneity (*I*²>90%), indicating that the pooled effect estimates should be interpreted with caution. A meta-regression to further explore potential study-level sources of this heterogeneity (e.g., baseline HbA1c, antidiabetic medication, structured Ramadan-focused education, body mass index, and fasting duration) was considered; however, several of these variables were inconsistently or incompletely reported across the included studies, precluding a reliable meta-regression analysis. Second, most of the included studies employed a before-after design without a consistent control group, limiting causal inferences regarding the effects of RF and leaving the findings susceptible to residual confounding factors. Third, the subgroup analyses were exploratory in nature and several subgroup strata included only a small number of studies, limiting their statistical power to definitively identify the sources of heterogeneity. Fourth, publication bias was detected for FBG and HbA1c levels, suggesting the presence of small-study effects that may have influenced the magnitude of the pooled estimates. Fifth, the literature search was restricted to English-language publications and did not include gray literature, raising the possibility that relevant studies were not identified during the selection process. Sixth, because the SDs of within-participant changes or pre-post correlation coefficients were not reported in most studies, a paired variance adjustment could not be performed. Consequently, the pooled analyses relied on the reported summary statistics before and after RF, which may have resulted in more conservative variance estimates. Future research should therefore prioritize more homogeneous prospective study designs, standardized reporting of clinical outcomes, adverse events, and individual participant data meta-analyses to better define the true effects of RF and identify the patient subgroups most likely to benefit from or be at risk during RF.

In conclusion, on average, RF was associated with improvements in glycemic control, as demonstrated by reductions in FBG and HbA1c levels, as well as improvements in selected lipid parameters, namely LDL and TG, but not TC or HDL levels. Nevertheless, the pooled estimates should be interpreted as average associations across highly heterogeneous real-world populations, rather than as evidence of a consistent or universal effect of RF. The substantial between-study heterogeneity, variable occurrence of hypoglycemia and hyperglycemia, and evidence of publication bias for FBG and HbA1c levels collectively indicate that the metabolic response to RF is strongly context-dependent. Clinically, RF may provide metabolic benefits for selected patients with well-controlled T2DM, but it cannot yet be considered universally safe or beneficial for all patients. Future studies should employ more homogeneous and rigorously controlled prospective designs with standardized reporting of the baseline clinical characteristics, antidiabetic treatment regimens, medication dose adjustments, fasting duration, dietary patterns, and physical activities. In addition, individual participant data meta-analyses will be valuable for identifying the patient subgroups most likely to benefit from or be at risk during RF.

## Figures and Tables

**Fig. 1.**
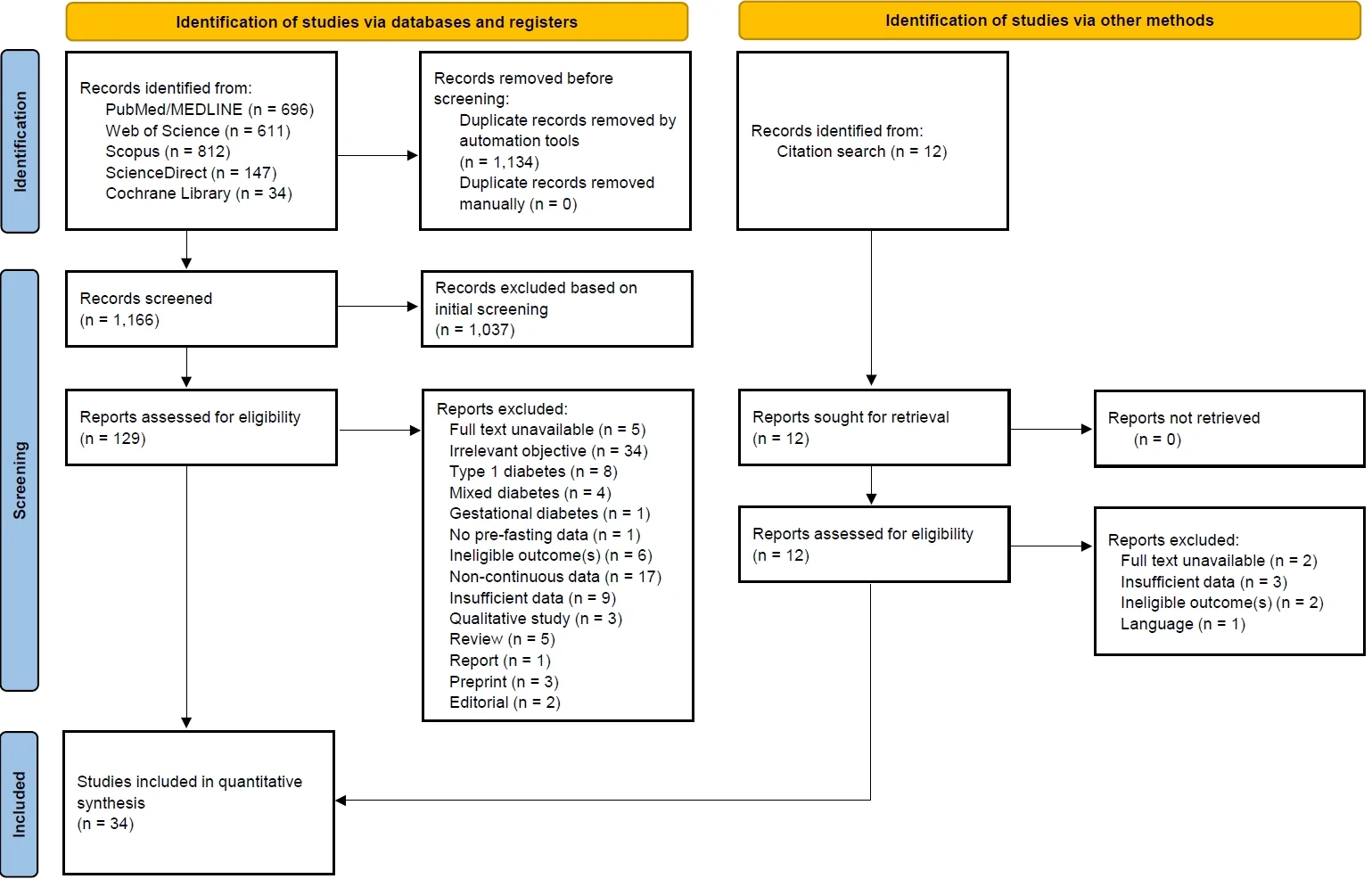
PRISMA (Preferred Reporting Items for Systematic Reviews and Meta-Analyses) flowchart of study selection process.

**Fig. 2.**
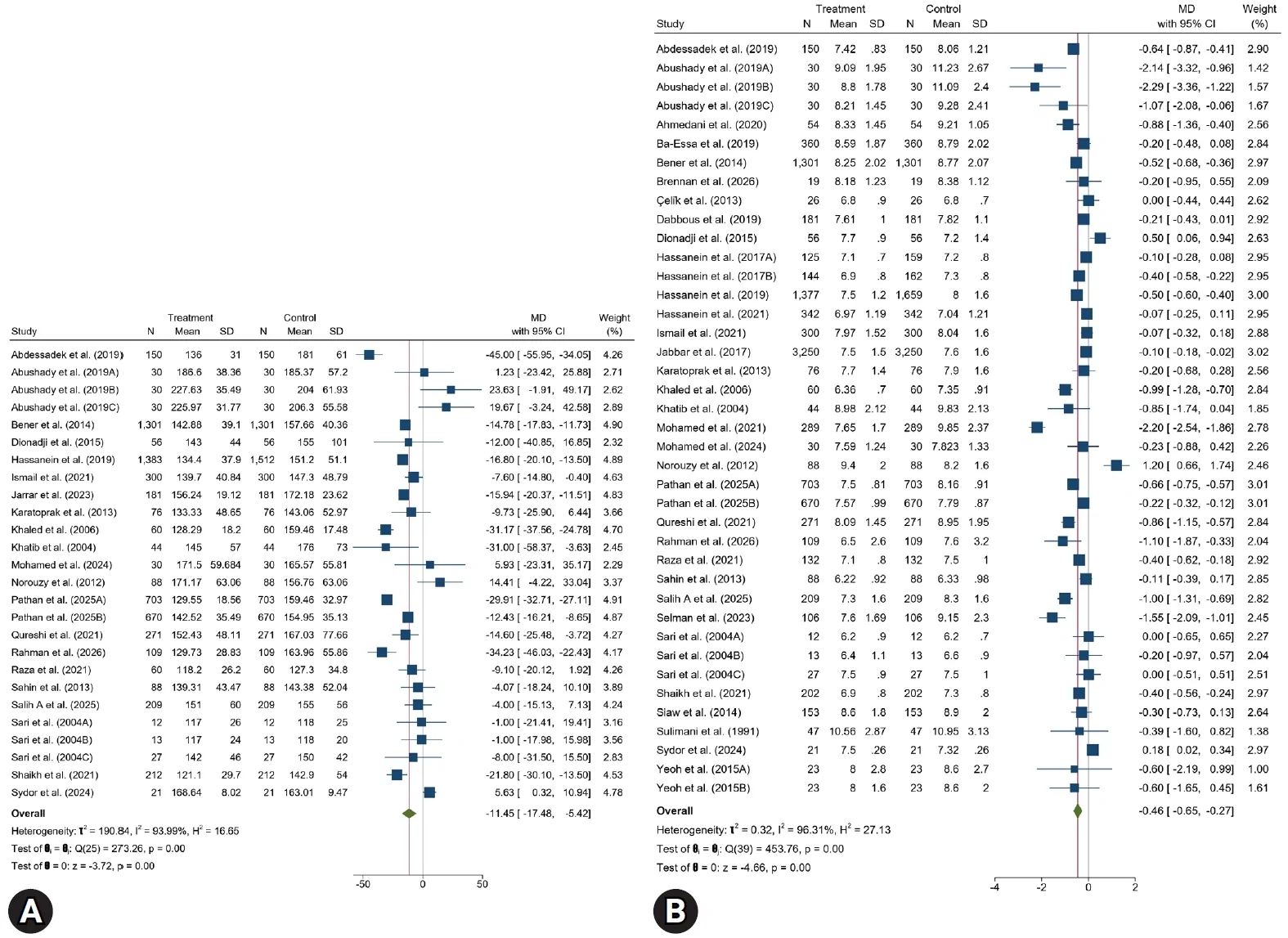
Forest plot of the effect of Ramadan fasting on glycemic control. (A) Fasting blood glucose (21 studies) and (B) HbA1c (33 studies). HbA1c, glycated hemoglobin; SD, standard deviation; MD, mean difference; CI, confidence interval.

**Fig. 3.**
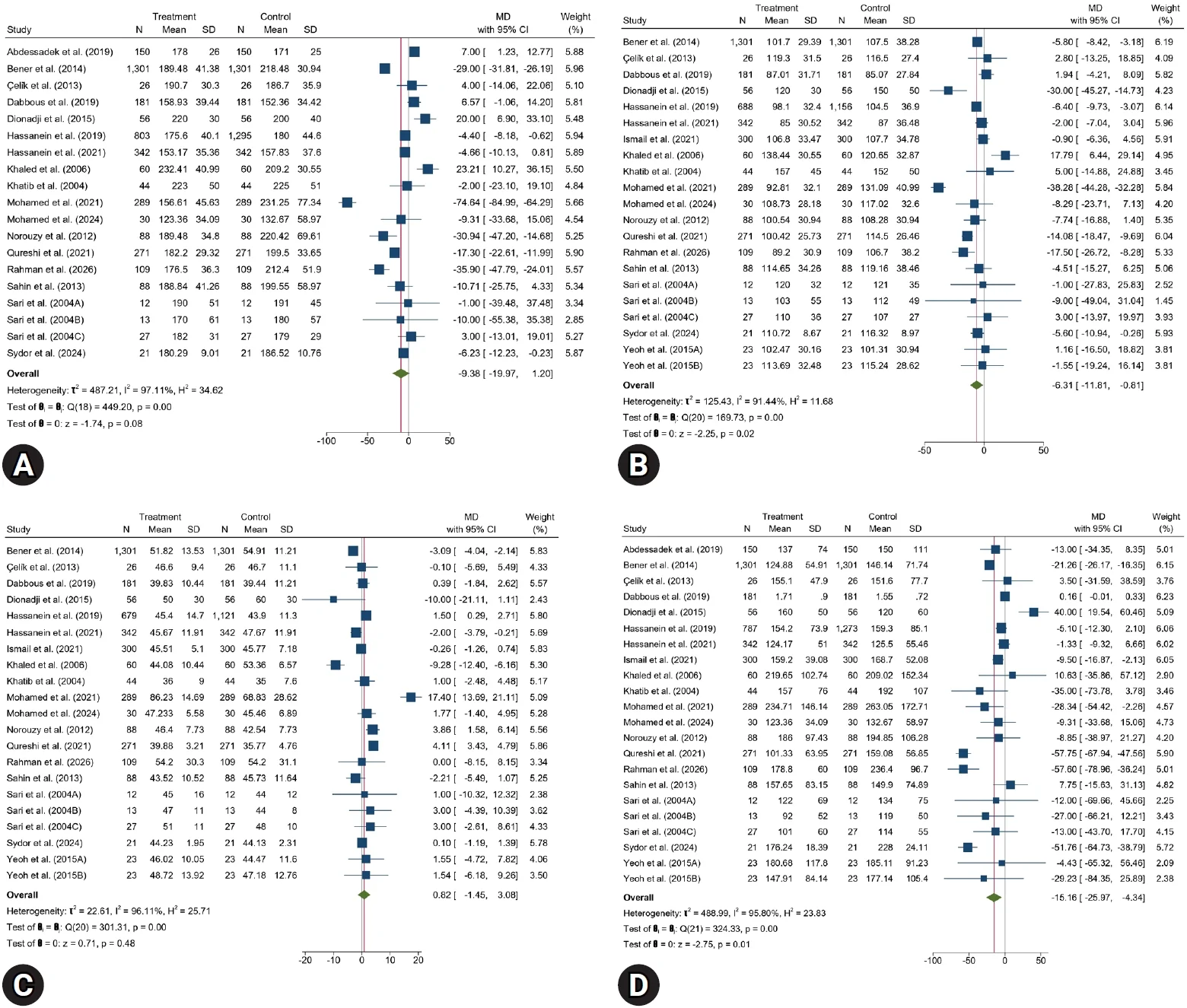
Forest plot of the effects of Ramadan fasting on lipid profiles. (A) Total cholesterol (17 studies), (B) low-density lipoprotein (18 studies), (C) high-density lipoprotein (18 studies), and (D) triglycerides (19 studies). SD, standard deviation; MD, mean difference; CI, confidence interval.

**Fig. 4.**
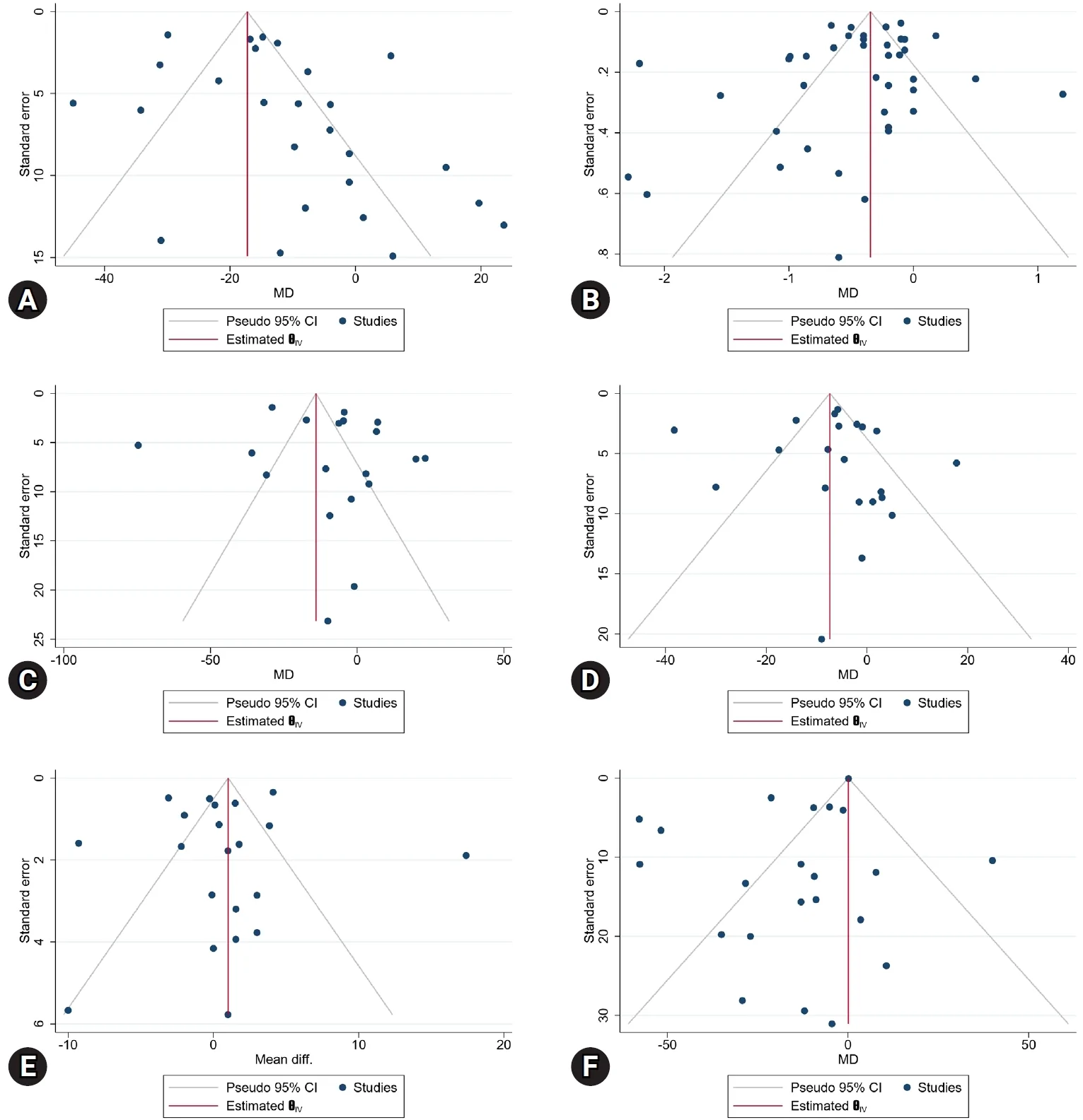
Funnel plots. (A) Fasting blood glucose, (B) HbA1c, (C) total cholesterol, (D) low-density lipoprotein, (E) high-density lipoprotein, and (F) triglycerides. HbA1c, glycated hemoglobin; MD, mean difference; CI, confidence interval.

**Fig. 5.**
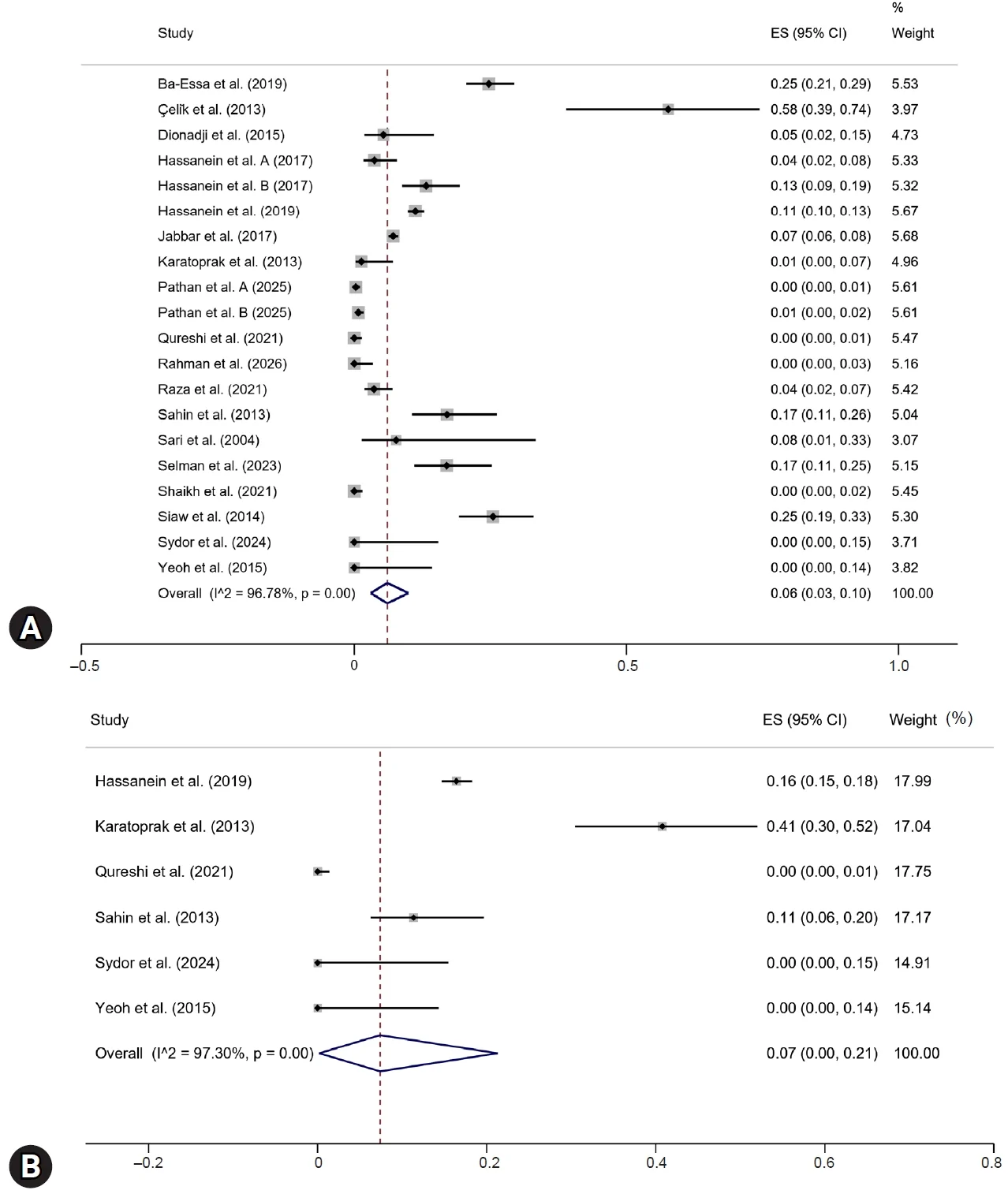
Meta-analysis of proportions for (A) hypoglycemia (18 studies) and (B) hyperglycemia (six studies). ES, effect size; CI, confidence interval.

**Table 1.** Study characteristics

Study	Country/region	Participants, n (% male)	Age (yr)	Patient characteristics	Ramadan fasting duration	Study quality
Abdessadek et al. [32]	Morocco	150 (38.0)	52, median	T2DM without major complications; antidiabetic medications; diabetes duration, 7.44±4.72 yr	NR	Good
Abushady et al. [33]	Egypt	Total: 90; Group A: 30 (80.0); Group B: 30 (33.3); Group C: 30 (66.7)	Group A: 48.67±9.06; Group B: 49.67±6.35; Group C: 49.03±6.56	T2DM with albuminuria and renal impairment (A), albuminuria alone (B), and neither (Group C); diabetes duration, 7.30–8.00 yr	Approximately 16 hr/day	Good
Ahmedani and Ghafoor [34]	Pakistan	54 (48.1)	54.65±9.32	T2DM receiving self-split insulin; diabetes duration, 13.83±6.27	NR	Fair
Ba-Essa et al. [35]	Saudi Arabia	360 (45.3)	53.8±9.3	T2DM; diabetes duration, 12.5±8.3 yr	Broken-fast days, 2.6±3.4	Fair
Bener and Yousafzai [36]	Qatar	1,301 (51.9)	45.9±15.3	T2DM receiving antidiabetic medication or insulin	NR	Good
Brennan et al. [37]	Bahrain	19 (68.0)	52±11.49	T2DM on stable antidiabetic medication	Entire Ramadan month	Good
Çeli̇k et al. [38]	Türkiye	26 (30.8)	57.8±7.0	T2DM for ≥1 year; HbA1c <7%; diabetes duration, 8.9±7.1 yr	26.8±4.6 days	Fair
Dabbous et al. [39]	Qatar	181 (63.5)	53.5±9.8	T2DM receiving ≥3 antidiabetic drugs; HbA1c ≤9.9%; diabetes duration, 10.6±6.5 yr	28 days, mean	Fair
Dionadji et al. [40]	Chad	56 (58.9)	50.11±9.8	T2DM; HbA1c ≤8%; mono- or combination therapy	NR	Good
Hassanein et al. [41]	Lebanon, Kuwait, and UAE	Total: 321; Group A: 159 (54.7); Group B: 162 (61.7)	Group A: 54.3±7.4; Group B: 52.3±7.7	T2DM; HbA1c ≤8.5%; sulfonylurea (A) or canagliflozin (B); diabetes duration, 6.5–7.6	Approximately 15 hr/day; 28.6±4.1 and 29.5±1.6 days	Good
Hassanein et al. [42]	Middle East and North Africa	1,749 (55.6)	55.2±11.1	T2DM receiving oral antidiabetic drugs with or without insulin; diabetes duration, 10.2±8.0 yr	15.8±1.0 hr/day; 27.7±5.0 days	Good
Hassanein et al. [43]	UAE	342 (52.3)	54	T2DM receiving oral antidiabetic drugs with or without insulin	NR	Good
Ismail et al. [44]	Egypt	300 (44.0)	53.23±8.79	Adults with T2DM	NR	Good
Jabbar et al. [45]	Middle East, Asia, North Africa, and Europe	3,250 (48.5)	56.9±10.7	T2DM from 13 countries; diabetes duration, 8.4±6.3 yr	Middle East, 27 days; Asia, 25.7 days; North Africa, 25.4 days; Europe: 21 days	Good
Jarrar et al. [46]	Palestine	181 (53.0)	NR	T2DM; HbA1c >6.5%	Entire month, 69.6%; partial month, 30.4%	Good
Karatoprak et al. [47]	Türkiye	76 (25.0)	57.4±10.1	T2DM; diabetes duration, 11.2±7.1 yr	16.5 hr/day	Good
Khaled et al. [48]	Algeria	60 (0)	51±10.0	Women with obesity and T2DM; diabetes duration, 5±2.5 yr	NR	Good
Khatib and Shafagoj [49]	Jordan	44 (100)	55±9.0	Men with T2DM receiving diet therapy, oral antidiabetic drugs, or insulin; diabetes duration, 8.37±7.02 yr	Approximately 13 hr/day	Good
Mohamed et al. [50]	Saudi Arabia	289 (60.6)	42.3±12.4	Adults with T2DM; diabetes duration >10 yr in 56.1%	≥12 hr/day	Good
Mohamed et al. [51]	Egypt	30 (20.0)	54.3±7.2	T2DM with overweight or obesity; antidiabetic medications; diabetes duration, 11.58±11.87 yr	Approximately 16 hr/day; 30 days	Good
Norouzy et al. [52]	Iran	88 (51.1)	51.3±10.6	T2DM receiving oral hypoglycemic drugs	13.33–14.45 hr/day; 25.17±0.67 days	Good
Pathan et al. [53]	Bangladesh	Total: 1,373 (40.6); Group A: 703 (39.7); Group B: 670 (42.1)	Group A: 50.21±11.11; Group B: 50.53±11.17	T2DM for ≥1 year; HbA1c <10%; ertugliflozin (A) or other oral antidiabetic drugs (B); diabetes duration, 6.26±4.98 yr	NR	Good
Qureshi et al. [54]	Bangladesh	271 (47.2)	48.65±10.6	T2DM; diabetes duration, 5.85±4.56 yr	NR	Good
Rahman et al. [55]	Bangladesh	109 (30.3)	40.7±12.8	T2DM receiving weekly tirzepatide with or without other antidiabetic drugs	NR	Good
Raza et al. [56]	Pakistan	220 (58.6)	50.9±10.3	Controlled or suboptimal controlled T2DM receiving gliclazide MR; diabetes duration, 3.1±3.8 yr	15.4±1.2 hr/day; 26.5±5.7 days	Good
Sahin et al. [57]	Türkiye	122 (32.8)	56.93±9.57	T2DM; diabetes duration, 5.73±5.1 yr	26.98±5.93 days	Good
Salih et al. [58]	Israel	209 analyzed (2011–2016 cohorts: 1,642–2,590)	56.9±6.3 to 61±6.6	T2DM for ≥1 year with available FBG and HbA1c data	NR	Fair
Sari et al. [59]	Türkiye	Total: 52; Group A: 12; Group B: 13; Group C: 27	Group A: 59±6.0; Group B: 57±5.0; Group C: 58±8.0	T2DM receiving diet therapy (A), sulfonylurea (B), or repaglinide (C); diabetes duration, 2.7–3.3 yr	NR	Good
Selman et al. [60]	Iraq	106 (49.1)	50±10.8	T2DM; diabetes duration, <10 in 62.3%	>25 days, 96.2%	Fair
Shaikh et al. [61]	India	246 (54.5)	53±10.6	Controlled or suboptimal controlled T2DM receiving gliclazide XR with or without other oral drugs; diabetes duration, 5.8±6.6 yr	13.9±1.9 hr/day; 29.1±1.7 days	Good
Siaw et al. [62]	Singapore	153 (37.3)	56.7±9.1	T2DM; diabetes duration, 13.2±9.1 yr	26±5 days	Good
Sulimani et al. [63]	Saudi Arabia	47 (59.6)	50.5±10.4	T2DM receiving metformin and/or glibenclamide	NR	Good
Sydor et al. [64]	Germany	21 (80.9)	61.1±1.8	T2DM receiving oral antidiabetic medication	Approximately 14 hr/day	Fair
Yeoh et al. [65]	Singapore	29 (52.0)	57±11	T2DM receiving oral antidiabetic medication with or without insulin; diabetes duration ≥10 yr in 60.7%	>15 days	Fair

T2DM, type 2 diabetes mellitus; HbA1c, glycated hemoglobin; FBG, fasting blood glucose; NR, not reported.

**Table 2.** Subgroup analysis

Outcome	df	MD (95% CI)	*p*-value of interaction	Heterogeneity	
				*I* ^2^	*p*-value
Fasting blood glucose					
Overall	25	–11.45 (–17.48 to –5.42)^a)^	NA	94%	<0.01
Subregion					
Northern Africa	6	–6.85 (–26.27 to 12.57)	0.57	94%	<0.01
Southern Asia	6	–16.61 (–26.99 to –6.23)^a)^		94%	<0.01
Western Asia	8	–11.32 (–15.99 to –6.65)^a)^		46%	0.16
Fasting duration (hr)					
<15	3	–7.03 (–27.04 to 12.97)	0.63	92%	<0.01
≥15	6	–1.34 (–12.99 to 10.31)		77%	<0.01
Study quality					
Fair	1	2.11 (–6.98 to 11.20)	<0.01	57%	0.13
Good	23	–12.73 (–18.99 to –6.47)^a)^		94%	<0.01
Glycated hemoglobin					
Overall	39	–0.46 (–0.65 to –0.27)^a)^	NA	96%	<0.01
Subregion					
Northern Africa	6	–0.93 (–1.51 to –0.35)^a)^	0.42	92%	<0.01
Southeastern Asia	2	–0.36 (–0.74 to 0.03)		0%	0.83
Southern Asia	7	–0.41 (–0.87 to 0.04)		98%	<0.01
Western Asia	17	–0.46 (–0.74 to –0.17)^a)^		94%	<0.01
Fasting duration (hr)					
<15	4	–0.41 (–1.52 to 0.70)	0.72	99%	<0.01
≥15	8	–0.63 (–1.03 to –0.23)^a)^		95%	<0.01
Study quality					
Fair	8	–0.50 (–0.90 to –0.11)^a)^	0.81	91%	<0.01
Good	30	–0.45 (–0.67 to –0.22)^a)^		97%	<0.01
Total cholesterol					
Overall	18	–9.38 (–19.97 to 1.20)	NA	97%	<0.01
Subregion					
Northern Africa	2	9.12 (–6.58 to 24.82)	<0.01	77%	0.03
Southern Asia	2	–26.76 (–39.09 to –14.44)^a)^		75%	0.01
Western Asia	9	–12.75 (–29.33 to 3.82)		97%	<0.01
Fasting duration (hr)					
<15	3	–28.83 (–61.85 to 4.19)	0.15	97%	<0.01
≥15	1	–4.51 (–8.25 to –0.78)^a)^		0%	0.70
Study quality					
Fair	2	0.58 (–8.81 to 9.98)	0.13	68%	0.03
Good	15	–11.49 (–23.83 to 0.84)		97%	<0.01
Low-density lipoprotein cholesterol					
Overall	20	–6.31 (–11.81 to –0.81)^a)^	NA	91%	<0.01
Subregion					
Northern Africa	2	3.17 (–11.30 to 17.65)	0.02	83%	0.01
Southeastern Asia	1	–0.19 (–12.69 to 12.31)		0%	0.83
Southern Asia	2	–13.61 (–17.25 to –9.97)^a)^		0%	0.31
Western Asia	9	–5.57 (–14.94 to 3.79)		92%	<0.01
Fasting duration (hr)					
<15	3	–12.67 (–31.00 to 5.66)	0.51	95%	<0.01
≥15	1	–6.48 (–9.74 to –3.23)^a)^		0%	0.81
Study quality					
Fair	4	–1.39 (–6.43 to 3.66)	0.13	27%	0.43
Good	15	–8.00 (–14.88 to –1.12)^a)^		94%	<0.01
High-density lipoprotein cholesterol					
Overall	20	0.81 (–1.45 to 3.08)	NA	96%	<0.01
Subregion					
Northern Africa	2	–2.54 (–9.08 to 4.00)	0.11	95%	<0.01
Southeastern Asia	1	1.55 (–3.32 to 6.41)		0%	0.99
Southern Asia	2	4.06 (3.41 to 4.72)^a)^		0%	0.61
Western Asia	9	1.75 (–2.15 to 5.66)		95%	<0.01
Fasting duration (hr)					
<15	3	5.50 (–2.27 to 13.26)	0.32	98%	<0.01
≥15	1	1.53 (0.40 to 2.66)^a)^		0%	0.87
Study quality					
Fair	4	0.23 (–0.84 to 1.30)	0.70	0%	0.99
Good	15	0.85 (–2.11 to 3.82)		97%	<0.01
Triglycerides					
Overall	21	–15.16 (–25.97 to –4.34)^a)^	NA	96%	<0.01
Subregion					
Northern Africa	3	–9.41 (–16.04 to –2.79)^a)^	0.15	0%	0.84
Southeastern Asia	1	–18.06 (–58.92 to 22.80)		0%	0.55
Southern Asia	2	–43.87 (–73.13 to –14.61)^a)^		85%	0.01
Western Asia	9	–9.78 (–19.12 to –0.43)^a)^		85%	<0.01
Fasting duration (hr)					
<15	3	–33.79 (–53.72 to –13.87)^a)^	0.01	60%	0.05
≥15	1	–5.44 (–12.34 to 1.47)		0%	0.74
Study quality					
Fair	4	–17.69 (–43.69 to 8.31)	0.83	89%	<0.01
Good	16	–14.47 (–26.63 to –2.31)^a)^		92%	<0.01

MD, mean difference; CI, confidence interval; df, degrees of freedom; NA, not applied.

a) *p*<0.05, statistically significant.

## Supplementary materials

Supplementary Table 1 and Supplementary Fig. 1 can be found at https://doi.org/10.12701/jyms.2026.43.52.

Supplementary Table 1. Search strategy

Supplementary Fig. 1. Leave-one-out sensitivity analyses: (A) fasting blood glucose, (B) HbA1c, (C) total cholesterol, (D) low-density lipoprotein, (E) high-density lipoprotein, and (F) triglycerides. HbA1c, glycated hemoglobin; MD, standard deviation; MD, mean difference; CI, confidence interval.
