# Supplementary material for: Effects of Ramadan fasting on glycemic control and lipid profile in type 2 diabetes mellitus: a systematic review and meta-analysis of clinical studies
Source: J Yeungnam Med Sci. 2026 Aug 5;43:52. doi: 10.12701/jyms.2026.43.52 (PMC13681063; doi:10.12701/jyms.2026.43.52)
Supplement: Supplementary file 1 [file jyms-2026-43-52-Supplementary-Fig-1.pdf]

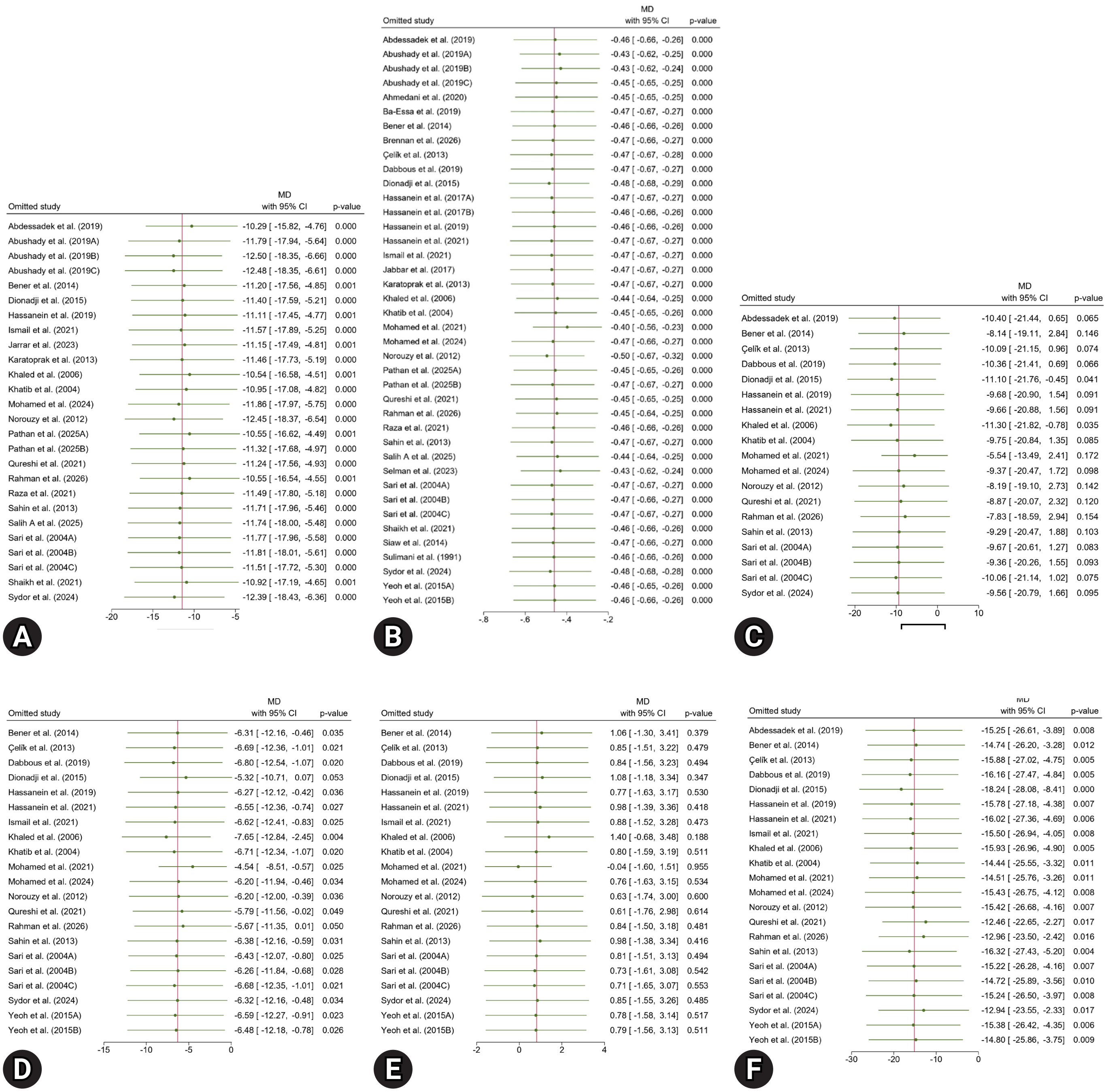

Supplementary Fig. 1. Leave-one-out sensitivity analyses: (A) fasting blood glucose, (B) HbA1c, (C) total cholesterol, (D) low-density lipoprotein, (E) high-density lipoprotein, and (F) triglycerides. HbA1c, glycated hemoglobin; MD, mean difference; CI, confidence interval.
