# Supplementary material for: Effects of Ramadan fasting on glycemic control and lipid profile in type 2 diabetes mellitus: a systematic review and meta-analysis of clinical studies
Source: J Yeungnam Med Sci. 2026 Aug 5;43:52. doi: 10.12701/jyms.2026.43.52 (PMC13681063; doi:10.12701/jyms.2026.43.52)
Supplement: Supplementary file 2 [file jyms-2026-43-52-Supplementary-Table-1.pdf]

Supplementary Table 1. Search strategy

Database	String
PubMed/MEDLINE	("Ramadan"[All Fields] AND ("fasted"[All Fields] OR "fasting"[MeSH Terms] OR "fasting"[All Fields] OR "fastings"[All Fields] OR "fasts"[All Fields])) AND (("diabetes mellitus, type 2"[MeSH Terms] OR "type 2 diabetes mellitus"[All Fields]) OR ("glycaemic control"[All Fields] OR "glycemic control"[MeSH Terms] OR ("glycemic"[All Fields] AND "control"[All Fields]) OR "glycemic control"[All Fields]) OR ("blood glucose"[Supplementary Concept] OR "blood glucose"[All Fields] OR "blood glucose"[MeSH Terms] OR ("blood"[All Fields] AND "glucose"[All Fields])) OR ("glycated hemoglobin"[All Fields] OR "hba1c"[All Fields] OR "glycated hemoglobin"[MeSH Terms] OR ("glycated"[All Fields] AND "hemoglobin"[All Fields]) OR "hba1cs"[All Fields]) OR ("lipid profile"[All Fields]) OR ("cholesterol"[Supplementary Concept] OR "cholesterol"[All Fields] OR "cholesterol"[MeSH Terms] OR "cholesterol s"[All Fields] OR "cholesterole"[All Fields] OR "cholesterols"[All Fields]) OR ("lipoproteins ldl"[All Fields] OR "low density lipoprotein"[All Fields] OR "lipoproteins, ldl"[MeSH Terms] OR ("lipoproteins"[All Fields] AND "ldl"[All Fields]) OR "ldl lipoproteins"[All Fields] OR ("low"[All Fields] AND "density"[All Fields] AND "lipoprotein"[All Fields])) OR ("lipoproteins hdl"[All Fields] OR "high density lipoprotein"[All Fields] OR "lipoproteins, hdl"[MeSH Terms] OR ("lipoproteins"[All Fields] AND "hdl"[All Fields]) OR "hdl lipoproteins"[All Fields] OR ("high"[All Fields] AND "density"[All Fields] AND "lipoprotein"[All Fields])) OR ("triglycerid"[All Fields] OR "triglycerides"[Supplementary Concept] OR "triglycerides"[All Fields] OR "triglyceride"[All Fields] OR "triglycerides"[MeSH Terms] OR "triglycerids"[All Fields]))
Web of Science	#1 Ramadan OR "Ramadan fasting" OR "Ramadan intermittent fasting"<br>#2 "type 2 diabetes mellitus" OR "diabetes mellitus" OR "glycaemic control" OR "glycemic control" OR "blood glucose" OR "glycated hemoglobin" OR "HbA1c" OR "lipid profile" OR cholesterol OR "low density lipoprotein" OR LDL OR "high density lipoprotein" OR HDL OR triglyceride*<br>#3 #1 (Topic) AND #2 (Topic)
Scopus	TITLE-ABS-KEY (Ramadan OR "Ramadan fasting" OR "Ramadan intermittent fasting") AND TITLE-ABS-KEY ("type 2 diabetes mellitus" OR "diabetes mellitus" OR "glycaemic control" OR "glycemic control" OR "blood glucose" OR "glycated hemoglobin" OR "HbA1c" OR "lipid profile" OR cholesterol OR "low density lipoprotein" OR LDL OR "high density lipoprotein" OR HDL OR triglyceride*) AND (LIMIT-TO (DOCTYPE , "ar")) AND (LIMIT-TO (LANGUAGE , "English")) AND (LIMIT-TO (SRCTYPE , "j"))
ScienceDirect	Title, abstract, keywords: (Ramadan OR "Ramadan fasting") AND ("type 2 diabetes mellitus" OR "diabetes mellitus" OR "glycemic control" OR "blood glucose" OR "glucose" OR "lipid profile" OR "lipid")
Cochrane Library	Title Abstract Keyword: (Ramadan OR "Ramadan fasting" OR "Ramadan intermittent fasting") AND ("type 2 diabetes mellitus" OR "diabetes mellitus" OR "glycaemic control" OR "glycemic control" OR "blood glucose" OR "glycated hemoglobin" OR "HbA1c" OR "lipid profile" OR cholesterol OR "low density lipoprotein" OR LDL OR "high density lipoprotein" OR HDL OR triglycerides)
